# A Technical Note of Improvement of the Elnady Technique for Tissue Preservation in Veterinary Anatomy

**DOI:** 10.3390/ani12091111

**Published:** 2022-04-26

**Authors:** Valentina Bernal, Pedro Aburto, Bárbara Pérez, Marcelo Gómez, Juan Claudio Gutierrez

**Affiliations:** 1 Institute of Pharmacology and Morphophysiology, Austral University of Chile, Valdivia 5090000, Chile; valentina.bernal@alumnos.uach.cl (V.B.); pedroaburto@uach.cl (P.A.); 2 Department of Anatomy, Physiology and Cell Biology, School of Veterinary Medicine, University of California, Davis, CA 95616, USA; jcgutierr@ucdavis.edu

**Keywords:** veterinary anatomy, teaching, preservation, Elnady technique

## Abstract

**Simple Summary:**

The Elnday technique is a method to preserve anatomical specimens. The aim of this study was to monitor different biological specimens after modifications to the Elnady technique. For the study, an equine heart, a canine heart, two Chilean frogs, and one canine specimen with thoracic and abdominal viscera were used. Results showed and good preservation of the organs and reactivation of the natural color of the specimens.

**Abstract:**

Teaching veterinary anatomy has been subjected to changes and restrictions that have promoted the development of new techniques for preserving organs and cadavers. The Elnady technique is a recent method for the conservation of tissues. Specimens produced with this technique are realistic, durable, soft, and flexible, but an undesirable feature is the discoloration of tissues. In the present study, we describe modifications of the Elnady technique for organ and tissue preservation. Specimens were prepared on the theoretical basis of the Elnady technique, but at low temperatures and with longer durations for the fixation, dehydration, glycerin impregnation and curing processes. Furthermore, the tissues were pigmented with a red vegetable pigment before dehydration or in the glycerin impregnation process. The results show high-quality specimens with minimal shrinkage and natural color aspects. The modified Elnady technique is adequate for producing specimens of better contrast for education purposes.

## 1. Introduction

In recent years, the teaching of anatomy has been subjected to changes and improvements. Additionally, there is a tendency to reduce the use of animals in teaching and research [1]. The veterinary medical curriculum has undergone changes such as a reduction in total hours of anatomy teaching, financial constrictions, and facility restrictions. Such limitations have translated into the use of new teaching formats with a smaller number of cadavers [2]. This situation has promoted the development of new techniques for the preservation of organs and cadavers. New preservation processes must meet safety, environmental, and cadaver use regulations.

Plastination, also called forced polymer impregnation, allows the preservation of tissues resulting in dry, durable, odorless, and life-like specimens [3,4]. The plastination process consists of four basic steps: fixation, dehydration, forced impregnation, and tissue curing or hardening. In the full process, the water and lipids of the biological tissue are replaced by polymers (silicone, polyester, epoxy) [3]. The plastination technique provides high-quality teaching specimens. Even though plastination offers many advantages, the plastinated tissue is rigid and lacks natural elasticity, which is a disadvantage for the teaching–learning process, including endoscopic observation of cadavers [5,6]. Recently, a new modified form of plastination emerged, the Elnady technique, developed by Dr. Fawzy Elnady in the Department of Anatomy and Embryology, Faculty of Veterinary Medicine, at Cairo University [7,8,9]. This technique improves plastination results in several aspects. The Elnady technique allows a conservation process at room temperature, removing the need for expensive plastination labs. The technique is inexpensive and does not require patented chemicals [8,9]. Specimens produced with the Elnady technique are realistic, durable, soft, and flexible. As flexibility is reduced in plastinated tissues, this represents a great advantage for the teaching–learning process. One pitfall of the Elnady technique of plastination is unwanted tissue discoloration, which is detrimental to the end result.

The objective of this study is to describe modifications to the Elnady technique. Such modifications allow for a more natural and realistic preserved biological specimen.

## 2. Materials and Methods

### 2.1. Specimens

For this study, one equine heart, one canine heart, a dog specimen of thoracic and abdominal viscera, and two Chilean frogs (*Caudiverbera gayi*) were used. Specimens did not show apparent trauma and/or degenerative or neoplastic/metastasic processes. This study was performed at the Veterinary Anatomy Unit of the Institute of Pharmacology and Morphophysiology, Faculty of Veterinary Sciences, Austral University of Chile, Chile. Specimens were obtained after euthanasia from cadaver donation programs. Chilean frogs were donated by a Bachelor of Science Student from the Austral University of Chile. The equine heart, the canine heart, and thoracic and abdominal viscera were cadaver donations from the Veterinary Teaching Hospital at the Austral University of Chile.

### 2.2. Fixation

All the specimens were submerged into an adequate fixation solution. The fixation solution was composed of 1 lt consisting of: 700 mL of distilled water, 100 mL of 37% formalin, 100 mL of 95% alcohol, 100 mL of glycerin, 10 g of thymol crystals and 1.5 mL of eucalyptus essence (approximately 30 drops) for a pleasant odor. The thymol crystals (fungicide agent) had previously been dissolved in alcohol. The solution was stirred for 10 min with a glass spoon. The glycerin was used as a humectant agent. The fixative/organ ratio was 5 times the original volume of the specimen. The samples were submerged into the solution and covered with cotton cloths to prevent floating. Specimens were fixed and stored in a cold room with a temperature range between 0 and −1 °C. The solution and the specimens were kept permanently in properly closed containers. The fixing time of the specimens varied between 15 and 60 days, depending on the volume (Table 1). Once the specimens were fixed, they were removed and washed with plenty of running tap water for 48 h. Next, dissections were performed. Excessive fat and connective tissue were removed from the specimens. For the heart, a ventricular anatomical flap was performed to observe the ventricular cavities and the internal structures, as well as cannulation of the blood vessels to prevent it from collapsing. Additionally, bleaching of the specimens was carried out with hydrogen peroxide of 5 volumes, for 24 to 48 h, depending on the volume of the specimen.

### 2.3. Staining of Specimens

Due to the formaldehyde darkening of tissues, the staining step was added for a more realistic appearance of the specimens. In this step, red vegetable pigments (pastry or gourmet dyes) diluted in water were used (3–9 pigment drops in 50 cc of distillate water). The dilution and the number of layers applied determined the intensity of tissue coloration. A brush or airbrush was used for better coverage of the specimen. For large specimens, it is recommended that the staining process be carried out in the glycerin impregnation step (see this step below), for a homogeneous staining and a decrease in working hours.

### 2.4. Acetone-Based Dehydration

This step was performed to remove water and lipids from tissues. Dehydration was performed after pigmentation was completed. The organs were submerged for 2 weeks in baths of 80% Acetone at −5 °C, followed by 2 weeks in 90% acetone at −5 °C, finishing with 100% acetone at room temperature (3–4 weeks or more), until the specimen reached 99% of acetone (measured with acetonometer). Each bath contained a volume of acetone 5–10 times the volume of the specimens. Acetone levels were checked every 2 to 3 days during the process with acetone replacements to maintain the desired concentrations. Cold dehydrations (80% and 90%) cause less tissue retraction. Dehydration at room temperature (100%) allows degreasing of the specimen. Dehydration was complete when water concentration measured less than 1%.

### 2.5. Glycerin Impregnation

Impregnation was performed once the dehydration process was finished. After rinsing the specimens from acetone, they were submerged in a bucket with glycerin. The bucket was appropriately closed with a lid. The volume ratio was 5 times the size of the specimen. The duration of the impregnation step was determined by the size and volume of the specimen (Table 1). If the staining process was performed in this step, the pigment was added directly to the glycerin and mixed until complete homogenization. The amount of pigment used was dependent on the volume of glycerin. Finally, the specimens were submerged to continue the process.

### 2.6. Curing

Once the previous process was finished, specimens were removed, drained for 2 to 10 days, and cleaned of excess glycerin with adsorbent paper. After that, specimens were stored in closed cotton bags. Cotton bags were placed in plastic buckets and covered with cornstarch powder for 1 to 3 weeks; in this period, the tissues were not in direct contact with the cornstarch. Specimens were left at room temperature until glycerin exudation was no longer observed. If a continuous exudation of glycerin was observed, a second curing process was performed. Once the curing process was finished, specimens were cleaned with brushes or air compressors if necessary. Next, specimens were stored at room temperature.

## 3. Results

**Figure 1:**
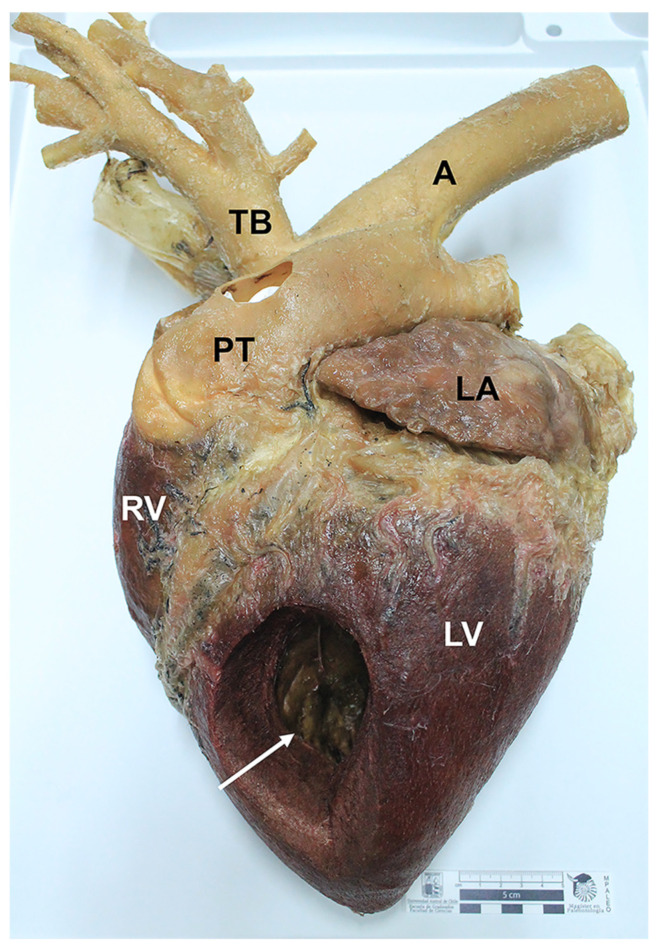
Photograph of the left side of an equine heart (*facies auricularis*) preserved and stained by the modified Elnady technique. A window opening in the left ventricle (white arrow). A: Aorta; LA: Left auricle; TB: Brachiocephalic trunk; PT: Pulmonary trunk; LV: Left ventricle; RV: Right ventricle. Red color in the ventricles is clearly observed in the organ.

**Figure 2:**
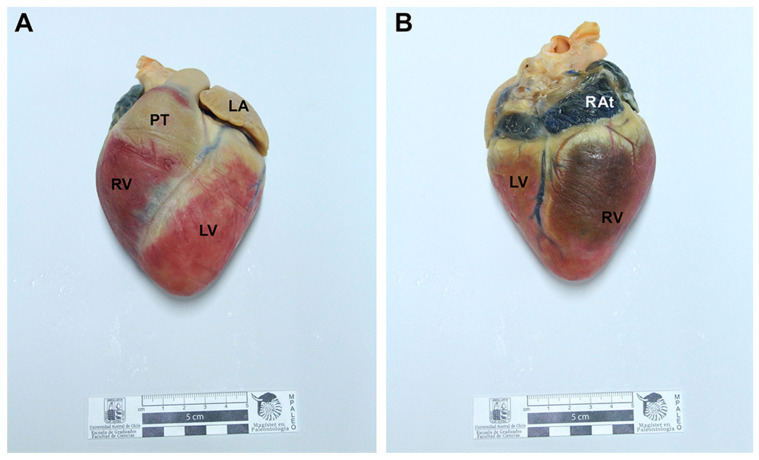
Photographs of the right side of a canine heart preserved and stained by the modified Elnady technique. (**A**) Left side (*facies auricularis*) of the heart. (**B**) Right side (*facies atrialis*) of the heart. LA: Left auricle; RAt: Right atrium; PT: pulmonary trunk, RV: Right ventricle; LV: Left ventricle. The ventricles were colored (red) by applying dying previous to the dehydration process. The coronary veins were injected with blue latex.

**Figure 3:**
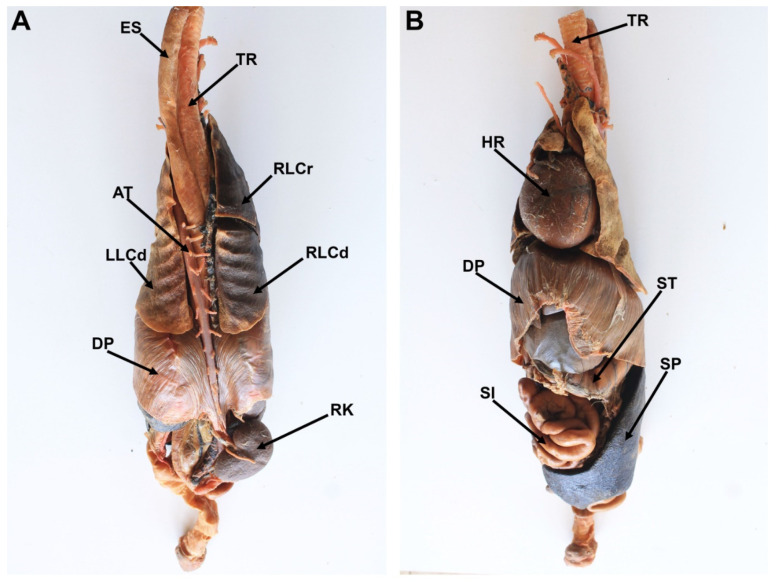
Photograph of the dorsal (**A**) and ventral (**B**) view of the canine thoracic and abdominal viscera prepared by the modified Elnady technique. ES: esophagus; TR: Trachea; AT: Thoracic Aorta; HR: Heart; RLCr: Cranial lobe of the right lung; RLCd: Caudal lobe of the right lung; LLCd: Caudal lobe of the left lung; DP: Diaphragm; ST: Stomach; RK: Right kidney; SI: Small intestine; SP: Spleen.

**Figure 4:**
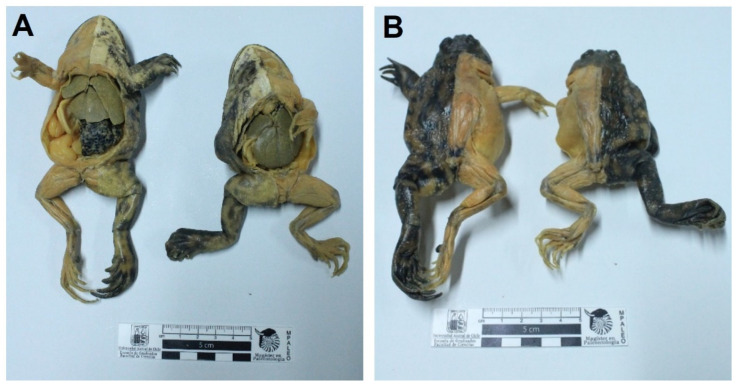
Photograph of two specimens of Chilean frog (*Caudiverbera gayi*), ventral (**A**) and dorsal (**B**) view, preserved by the modified Elnady technique. These specimens were not pigmented. Notice that the musculature and organs are pale and unnatural.

Final specimens were durable, soft, flexible, and odorless. The length for the full process was 16 to 41 weeks (Table 1). Neither shrinkage nor distortions were observed. The external and internal anatomical features of the specimens were adequately preserved. Regarding the equine and canine hearts, structures such as the left auricle, left ventricle, right ventricle, right auricle, aorta, brachiocephalic trunk, and pulmonary veins were adequately preserved (Figure 1). Anatomical details such as coronary arteries were clearly delineated and recognized. Final heart specimens displayed a natural red color in the ventricles, and a clear distinction between auricles (Figure 1 and Figure 2). For the thoracic and abdominal viscera of the dog, pigmented by the glycerin immersion method, natural and homogeneous coloration of the tissues was obtained, with excellent contrasts between the main organs (esophagus, trachea, lungs, thoracic aorta, diaphragm, stomach, kidneys, small intestine, and spleen) (Figure 3). Chilean frogs were not pigmented to demonstrate differences with the pigmented specimens. The musculature and lungs displayed an unnaturally pale color, affecting the quality of the preparation (Figure 4). Nevertheless, anatomical structures such as the lungs, liver, stomach, small intestine, and thoracic and pelvic limb musculature were adequately preserved. Results of this study reveal that specimens stained by the two methods displayed a good and natural color aspect for educational purposes.

The proposed modification of the Elnady technique was low-tech and reduced in start-up cost. The cost for this study varied according to the size of specimens. The fixation process varied from 30 to 200 USD, for dehydration, from 72 to 500 USD, for impregnation, from 38 to 450 USD, and for curing, from 12 to 112 USD.

## 4. Discussion

The modification to the Elnady technique consisted of adaptions to the steps of fixation, dehydration, and glycerin impregnation, as well as adding a pigmentation step to give aesthetic value to the specimens. In some parts of this protocol, the times the specimens remained at each step were extended. The Elnady technique establishes a time range of 7–8 weeks for specimen processing [7,8]. The present study has extended that time to 3–6 months. This time extension does not have any negative impact, but conversely, it allows for high-quality specimens.

Tissue fixation protocols can vary widely between laboratories, generally based on the characteristics of the specimens and the experience of the technician [10,11]. The rationale for longer times used in this study is related to the fact that specimens were not fixed by injection methods, and were not dissected before fixation. This allowed complete penetration of formaldehyde into the tissues. Studies by Elnady (2016) and El-Shafy et al. (2022) [8,12], proposed a fixation of cadavers immediately after euthanasia by bleeding, canalization, and subsequent injection of formaldehyde. Although this method speeds the fixation process up, it can only be used in animals that are donated alive, which in practice is not common. On the other hand, Elnady (2016) and Shafyet (2022) fixed the specimens at room temperature [8,12]. In the present study, the fixation process was performed in a cold room (+1 to +5 degrees centigrade) to delay the decomposing process in large specimens and to allow color impregnation [13]. In addition, a decreased exposure to formaldehyde by the anatomy staff was possible due to such adjustments. In the present study, acetone-based dehydration, described in the plastination protocol, was used. This protocol has been considered the most successful method for the dehydration of tissues reported in the literature [14]. Several studies have reported that warm (room temperature) acetone caused excessive shrinkage and incomplete dehydration of the specimens [14,15,16]. In the present study, dehydration was performed in consecutive concentrations of 80% and 90% at lower temperatures (−5° to −10 °C), and finished with 100% acetone at room temperature, to facilitate the degreasing of the specimen. These modifications resulted in specimens with minimal shrinkage, which is optimal for teaching purposes. Studies have indicated that dehydration using cold acetone (freeze-substitution) results in minimal shrinkage, and reduced explosive risks of this hazardous solvent [14].

In this study, longer times of impregnation with glycerin resulted in a greater flexibility of specimens compared to the short times described in previous Elnady protocols [8,12]. This is a desirable feature, especially for preserved tubular organs and biomechanics concepts. Additionally, there are factors associated with the type of sample and the environmental conditions that affect the extent of the process. Large organs (i.e., horse heart) require a longer impregnation time given the size and thickness of their muscular walls. Another factor is the environmental temperature, since at higher temperatures (summer, 25–30 °C), the impregnation process will be faster than at lower temperatures (winter, 10–18 °C). This occurs because glycerin viscosity has an inverse relationship with temperature, significantly affecting the impregnation times. Therefore, it is suggested to control the environmental temperature during sample preparation.

The colorizing material used in this study has not been described previously in classical or modified protocols of the Elnady technique [8,12]. Staining allowed an enhanced, natural external organ appearance, helping to dilute the grey color of the formalin-fixed specimens. The coloration of the specimens can be obtained by the injection of a colorant material in the vascular system and/or by incorporation of dyes or coloring agents before the fixation process or after the fixation process [17,18,19,20]. Chemicals and dyes used in plastination techniques are patented, have a high cost, and often are not available [8]. In the present study, a natural, non-expensive dye was applied directly to the specimen with a brush before the dehydration step. Another alternative is to stain the specimens during the immersion in glycerin. This method is simple, and adequate results were achieved in isolated organs. However, a disadvantage of this method is that red pigments are not selective and penetrate all tissues. Therefore, the desired contrast of muscle, cartilage, tendons, and aponeuroses could not be achieved properly. Therefore, for musculoskeletal structures, it is preferable to use the vascular injection method (i.e., colored epoxy) [17]. In the classical Elnady technique, the specimens are injected with colored latex, which is an excellent method to mark the main blood vessels. However, due to the high density of latex, it is not possible to reach the capillary level to achieve a complete and natural coloration of the tissues [8,12,21].

The curing step was performed using cotton cloth bags since the specimen exudes large amounts of glycerin. This will cause the cornstarch to adhere to the cotton fabric. In the present study, the curing was not performed in direct contact with the cornstarch. Cornstarch residues can alter the appearance of the specimen. Furthermore, the use of cotton cloth bags reduces the amount of work required to clean up the cornstarch attached to the specimen. This is particularly important when processing organs that have natural folds (such as the intestine and stomach).

The Elnady technique has been used adequately in the veterinary sciences for neuroanatomical specimens, upper respiratory tract endoscopy in horses, exploration of reproductive organs, and teratology [7,9,18,21]. This technique allows for the creation of realistic, durable, soft, flexible, clean, dry, and odorless specimens [7,8,9]. The goal of this study was to describe modifications to the original technique that can help to improve the natural appearance of the specimens. The Elnady technique is relatively inexpensive and allows small anatomy laboratories to prepare specimens for teaching and display purposes. The results of the present study highlight that cadaver preservation techniques are not rigid; they can be updated and adapted to the conditions and reality of each anatomy laboratory. The results of the present study also suggest that our modifications of the Elnady technique are adequate for producing specimens with clear and distinct gross anatomical features with color reactivation, improving the anatomical teaching/learning experience. This technique can be performed at a low cost without the need for special facilities or expensive equipment, and can be implemented in any veterinary anatomy laboratory.

## 5. Conclusions

The results of the present study suggest that the modified Elnady technique is adequate for producing high-quality anatomical specimens with clear/distinct gross anatomical features and with color reactivation. The technique is easy to perform and requires low-cost materials.

## Figures and Tables

**Table 1:** Steps and duration of Elnady Technique process required to prepare 4 biological specimens; 1 equine heart, 1 canine heart, 2 Chilean frogs, and 1 canine specimen with thoracic and abdominal viscera.

	Time			
Steps	Equine Heart	Canine Heart	Chilean Frogs	Canine Thoracic and Abdominal Viscera
Dissection and fixation	60 days *	30 days	15 days	60 days
Pigmentation	1 day	1 day	1 day	3 days
Acetone-based dehydration				
80% acetone at −5 °C	3 weeks	2 weeks	2 weeks	4 weeks
90% acetone at −5 °C	3 weeks	2 weeks	2 weeks	4 weeks
99% acetone at room temperature	6 weeks	4 weeks	3 weeks	7 weeks
Glycerin impregnation	12 weeks	8 weeks	6 weeks	16 weeks
Curing				
-Draining	5 days	2–3 days	2–3 days	10 days
-Finishing	3 weeks	2 weeks	1 week	6 weeks

* Adult specimens.

## Data Availability

Data can be made available upon reasonable request to the authors.
